# Mitochondrial DNA Mutation Analysis in Breast Cancer: Shifting From Germline Heteroplasmy Toward Homoplasmy in Tumors

**DOI:** 10.3389/fonc.2020.572954

**Published:** 2020-10-27

**Authors:** Carlos Jhovani Pérez-Amado, Hugo Tovar, Laura Gómez-Romero, Fredy Omar Beltrán-Anaya, Verónica Bautista-Piña, Carlos Dominguez-Reyes, Felipe Villegas-Carlos, Alberto Tenorio-Torres, Luis Alberto Alfaro-Ruíz, Alfredo Hidalgo-Miranda, Silvia Jiménez-Morales

**Affiliations:** ^1^Laboratorio de Genómica del Cáncer, Instituto Nacional de Medicina Genómica, Mexico City, Mexico; ^2^Programa de Doctorado, Posgrado en Ciencias Bioquímicas, Universidad Nacional Autónoma de México, Mexico City, Mexico; ^3^Genómica Computacional, Instituto Nacional de Medicina Genómica, Mexico City, Mexico; ^4^Laboratorio de Investigación en Epidemiología Clínica y Molecular, Facultad de Ciencias Químico-Biológicas, Universidad Autónoma de Guerrero, Chilpancingo, Mexico; ^5^Instituto de Enfermedades de la Mama, FUCAM, Mexico City, Mexico

**Keywords:** mitochondrial DNA, breast cancer, mutations, heteroplasmy, haplogroups, molecular subtypes

## Abstract

Studies have suggested a potential role of somatic mitochondrial mutations in cancer development. To analyze the landscape of somatic mitochondrial mutation in breast cancer and to determine whether mitochondrial DNA (mtDNA) mutational burden is correlated with overall survival (OS), we sequenced whole mtDNA from 92 matched-paired primary breast tumors and peripheral blood. A total of 324 germline variants and 173 somatic mutations were found in the tumors. The most common germline allele was 663G (12S), showing lower heteroplasmy levels in peripheral blood lymphocytes than in their matched tumors, even reaching homoplasmic status in several cases. The heteroplasmy load was higher in tumors than in their paired normal tissues. Somatic mtDNA mutations were found in 73.9% of breast tumors; 59% of these mutations were located in the coding region (66.7% non-synonymous and 33.3% synonymous). Although the *CO1* gene presented the highest number of mutations, tRNA genes (T,C, and W), rRNA 12S, and *CO1* and *ATP6* exhibited the highest mutation rates. No specific mtDNA mutational profile was associated with molecular subtypes of breast cancer, and we found no correlation between mtDNA mutational burden and OS. Future investigations will provide insight into the molecular mechanisms through which mtDNA mutations and heteroplasmy shifting contribute to breast cancer development.

## Introduction

Breast cancer is the most common cancer type in women worldwide (1); in 2018, 2.09 million cases and 627,000 deaths were estimated around the world (2). In Mexico, this malignancy is currently the leading cause of cancer-related deaths in women (3–5). Breast cancer is a heterogeneous disease in terms of the molecular features and clinical outcome; knowledge of the genetic causes is still incomplete. To comprehensively address this issue, the analysis of nuclear genes has been frequently done (4, 6–10). However, mutations in mitochondrial genes have also been reported to play important roles in human cancer development (11). Experimental evidence in mouse models demonstrated that G13997A and 13885insC mutations in the reduced form of the nicotinamide adenine dinucleotide dehydrogenase subunit 6 (*ND6*) gene are associated with the transition of poor metastatic tumor cells into a highly metastatic phenotype (12).

Mitochondria are indispensable for numerous cellular processes, such as respiratory energy metabolism, cell proliferation, apoptosis, senescence, and immune response (13). Mitochondrial DNA (mtDNA) is a circular DNA molecule of 16,569 bp containing 2 rRNA, 22 tRNA, and 13 genes encoding protein subunits for the mitochondrial complexes of the oxidative phosphorylation system (OXPHOS). The mitochondrial genome is characterized by many DNA molecules per cell and a high mutation rate that creates a state known as heteroplasmy (a mixture of mutant and normal mtDNA in cells) (14, 15). The relative abundance of certain *de novo* mutations might be pathogenic, although the heteroplasmic threshold effect and the molecular mechanisms underlying the mutation selection during disease development have not yet been established (15). However, the association among several mtDNA germline variants and a broad spectrum of human malignancies has been widely documented (16–18). For instance, the T16189C (D-Loop) and G10398A (A114T at *ND3* gene) germline variants have been commonly associated with an increased risk of cancer (17, 19–21). The important role of germline mtDNA variants in tumors has been noted in groups of variants inherited without any recombination, called haplogroups, conferring risk for cancer and as modifiers for potential metastasis and for the response to treatment in affected patients (22).

Genomic evidence suggests that mtDNA germline variants and tumor mutations are also involved in breast cancer development. mtDNA mutations have been reported in almost 60% of breast tumors, varying in nature (mainly single base substitutions and indels) and heteroplasmy levels (23–26); however, whether these mutations are drivers or passengers remains largely uncharacterized (27, 28). mtDNA sequencing from matched normal–tumor breast tissues has shown that a high proportion of the somatic mutations are singletons arising in a single patient and that many of those were under clonal expansion in the primary or metastatic tumors (17, 22, 28–33). To explore the landscape of mtDNA mutations in breast cancer from Mexican women and to know if mtDNA mutations and haplogroups are associated with the molecular subtypes of breast tumors or with overall survival (OS), we performed whole mtDNA sequencing in matched-paired primary breast tumors and peripheral blood.

## Materials and Methods

### Patient Data

We included matched tumor samples and peripheral blood from 92 women with breast cancer. Patients were recruited from the Instituto de Enfermedades de la Mama FUCAM (Mexico City, Mexico), and all patients provided written informed consent. Patients with chemotherapy, radiotherapy, and neoadjuvant treatment before the surgery were excluded. Based on the current international criteria and using immunohistochemical (IHC) markers, breast tumors were classified as luminal A (LA), luminal B (LB), HER2-positive (H2), and triple negative (TN) (34). Patients were followed up for at least 30 months (30–124) after the clinical diagnosis.

### Sample Processing and DNA Extraction

Peripheral blood was obtained before surgical treatment using EDTA vacutainer tubes, and the buffy coat (leukocytes) was separated out by centrifugation at 1850 × *g*. Fresh tumor tissues were obtained during surgical treatment and stored at −80°C until further analysis. Breast tumor samples with >80% of tumor cells were included. Total DNA from leukocytes and tumor samples were extracted using a Maxi Kit (Qiagen, Hilden, Germany) and AllPrep DNA/RNA Mini Kit (Qiagen, Hilden, Germany), respectively, according to the manufacturer’s instructions. The nucleic acids were quantified by spectrophotometry (NanoDrop, Thermo Fisher Scientific, Waltham, MA, United States) and stored at −20°C until library preparation.

### Whole mtDNA Sequencing

To avoid contamination with nuclear mitochondrial segments, the whole mtDNA was enriched using long PCR with two overlapping primer sets (Supplementary Table 1) and subsequently fragmented by sonication. Indexed paired-end mtDNA libraries were generated with the Nextera DNA Flex Library Prep Kit (Illumina, San Diego, CA, United States) according to the manufacturer’s instructions. Libraries were sequenced on an Illumina MiSeq using 150-bp paired-end read chemistry and 300 cycles (MiSeq Reagent Kit v2, Illumina, San Diego, CA, United States).

### Bioinformatic Analysis

We included mitochondrial genome data with depth coverage with at least 300X *per* base. A quality control (QC) evaluation was performed on the raw sequence data using the FastQC algorithm (35), and reads with Phred quality >25 were accepted for further analysis. The Burrows–Wheeler alignment (BWA-MEM) algorithm was used to align all sequences against the revised Cambridge Reference Sequence (GRCh38, GCF_000001405.38) (36). Duplicated reads on mtDNA were removed. Then, reads were realigned and quality scores were recalibrated using Genome Analysis Toolkit (GATK) (37). Haplogroups were assigned on the mtDNA variants detected in blood samples and using Haplogrep software (v2.1.19) and Phylotree databases (38, 39). The Strelka2 algorithm with default parameters (40) was used to identify somatic mutations by comparing germline and matched mtDNA variants. Only variants labeled PASS were considered high-quality variants (40). To reduce false positives, a somatic mutation was considered when the mutant allele fraction (MAF) was <0.001 in normal tissue and >0.01 in tumor tissue in all samples where it occurred. Germline variants were defined if MAF > 0.01 in the blood sample regardless of the proportion in tumors and if this occurred in at least one patient. Variants not fulfilling these criteria were considered as probable mutations. These thresholds were estimated based on the depth coverage. All variants displaying mutant allele abundance from 1% to <95% were deemed heteroplasmic. Homoplasmic was defined as a mutant allele >95% (32). Mutations were reviewed in the MITOMAP database to determine if they had been reported in breast cancer or in other malignancies (41). To predict their role as a driver or passenger, we used the Variant Effect Predictor (VEP) software with the default parameters (42). To assess whether an mtDNA profile was associated with the tumor subtype, we performed an unsupervised hierarchical clustering analysis accounting for all mtDNA mutations using R (version 3.6.0). The distribution of mtDNA somatic mutations among breast cancer subtypes was visualized using Venn diagrams (43).

### Statistical Analysis

Mutations were counted according to gene distribution, and frequencies were obtained for all analyzed patients. To assess the association between mtDNA mutations and haplogroups and clinical and molecular characteristics among groups, chi-square and Fisher’s exact tests were calculated when appropriate. Mann–Whitney *U* tests were used to evaluate differences in heteroplasmy levels between the blood and tumor tissues. An OS analysis was performed, including patients who were followed up to 124 months after diagnosis and initial treatment, defining OS as the time between initial diagnosis and death. mtDNA mutational burden was stratified into two groups: patients without any mutation and patients with mutations, as well as according to the mutational burden mean (patients with a low or high mutational burden). To evaluate whether somatic mtDNA mutational burden is associated with patient outcome, Kaplan–Meier survival curves were obtained and the hazard risk was estimated using a Cox proportional hazard model adjusting for variables that have been reported to influence the OS (age, tumor stage, clinical stage, and hormone receptor status). *P*-values < 0.05 were considered statistically significant. Statistical analysis was conducted using SPSS (Version 25, IBM Corp., Armonk, NY, United States). Plots were generated using R (version 3.6.0).

## Results

### Study Population

We included 92 unrelated patients with a mean age of 53.83 ± 11.5 years (range 33 to 92 years; Supplementary Table 2). The tumor subtype was assigned by IHC markers in 90 patients. Luminal A (63.3%) was the most frequent subtype, followed by LB (24.4%), TN (6.7%), and H2 (5.6%) subtypes.

### Distribution of Haplogroups

From 564 germline variants identified in the normal tissues, 296 were used by Haplogrep for haplogroup typing. After haplotyping the mtDNA of all patients, haplogroup A was the most frequent (44.6%), followed by haplogroups B (22.8%), C (11.9%), D (12%), and L (5.4%). Haplogroups H and J were found in 2.2 and 1.1% of patients, respectively. Stratification analysis by IHC subtypes revealed that haplogroups C and D were present only in LA and LB subtypes, and TN tumors carried haplogroups A and B. No statistically significant differences in the haplogroup distribution was found among the tumor subtypes (*P* > 0.05).

### Mitochondrial DNA Variant Distribution

The depth coverage of mtDNA for the examined samples was from 300× to 6000× *per* base (Supplementary Figure 1). 3404 variants carried by normal tissue (located in 564 positions across the mtDNA) and 3876 variants identified in tumor samples passed all quality controls. Blood samples ranged from 16 to 75 variants (X̄ = 37 ± 9), whereas tumor samples carried from 24 to 89 variants (X̄ = 42 ± 11).

**FIGURE 1:**
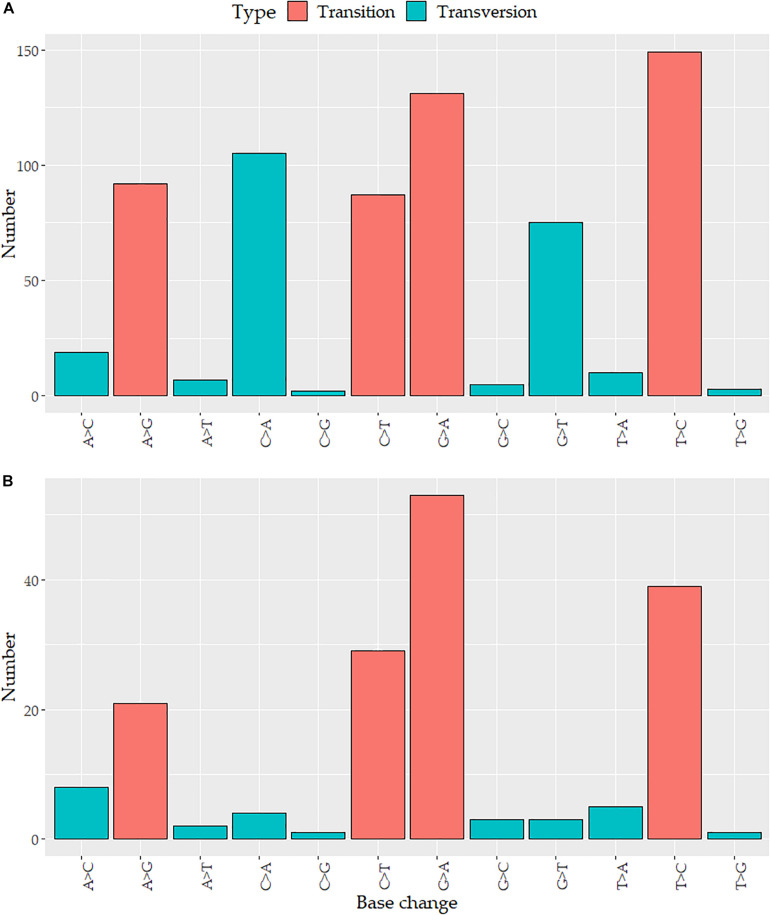
Distribution of nucleotide substitutions in the mitochondrial genome: the number of the substitution according to the base change in **(A)** tumor variants and **(B)** somatic mutations.

Variants detected in breast tumor tissues were located in 709 positions across the mtDNA, 685 (96.6%) of these variants were single-nucleotide variants (SNVs), 12 (1.7%) were small deletions, and 12 (1.7%) were small insertions. The variants were distributed around all mtDNA: 438 (61.8%) were located in coding genes (*ND5*: 69, *CO1*: 57, *ND4*: 50, *ND1*: 47, *CYB*: 43, *ATP6*: 40, *ND2*: 39, *CO3*: 25, *CO2*: 21, *ND3*: 17, *ND6*: 17, *ATP8*: 9, and *ND4L*: 7) and 271 (38.2%) in non-coding regions (D-Loop region: 114, rRNA: 101, tRNA: 47, and intergenic regions: 9). Given the nature of the mtDNA molecules, three variants were overlapped in the *ATP8* and *ATP6* genes. The mutation rate of mtDNA per kilobase (kb) in tumors was 42.79 variants/kb (vars/kb). Genes showing the highest number of vars/kb were *tRNA*-C (92.3), *tRNA*-T (92.3), *tRNA*-L1 (67.6), *tRNA*-W (59.7), *ATP6* (58.8), *12S* (51.4), *ND1* (49.2), and the D-Loop region (95.4 vars/kb) (Supplementary Figure 2). None of the 709 variants were detected in *tRNA*-A, *tRNA*-D, or *tRNA*-S1. Transitions and transversions accounted for 67% (459) and 33% (226) of the SNVs, respectively (Figure 1A).

Of the total variants detected in tumor tissues, 324 were defined as germline variants and 173 as somatic mutations. Two hundred eleven variants were considered as probable mutations.

### Germline Variants Are Highly Heterogeneous in the Heteroplasmic Levels in Breast Tumor Tissue

One hundred sixty-three (50.3%) germline variants were detected in more than two cases, and 161 (49.7%) were singletons, arising in only one patient. Only 15 out of 324 (4.6%) were identified in more than 10 (>11%) patients: T146C, A153G, A235G, A663G, A1736G, T4248C, A4824G, G8027A, C8794T, C16111T, T16189C, C16290T, G16319A, T16362C, and T16519C, with A663G (19.6%) being the most frequent germline variant.

**FIGURE 2:**
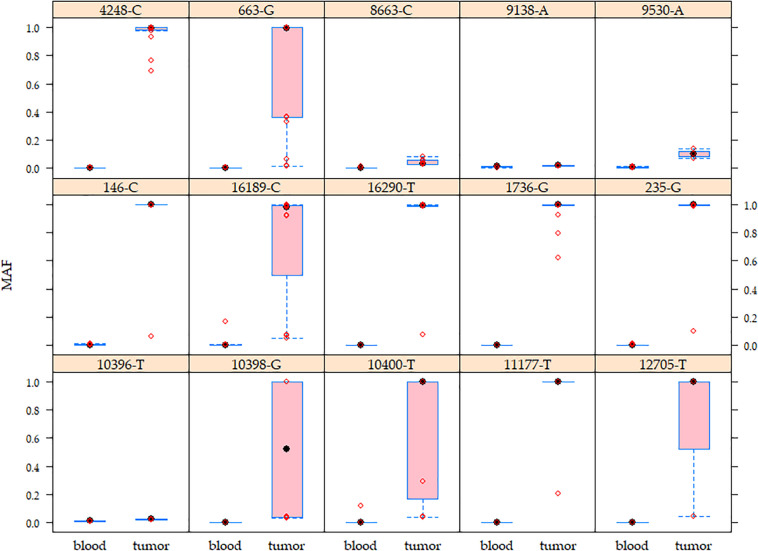
Mutant allele frequency (MAF) of the mitochondrial germline variants in matched peripheral blood–tumor tissues. Higher MAF variability was observed in breast tumors than in blood samples. The boxes show the MAF values for each variant. Red circles represent patients. These germline variants were detected from 4.3 to 19.6% of the analyzed population.

Heteroplasmy levels differed among tissues (blood <20%, tumors >1% and <95%) (Supplementary Figure 3). 127/324 (39.2%) germline variants exhibited a very low heteroplasmy level (1–2%) in both tissues, of which 42 (33.1%) were identified in more than two patients and the remaining 85 (66.9%) were singleton germline variants. To note, 25 (29.4%) of the singleton germline variants are reported in the MITOMAP database. Regarding the non-singleton germline variants, 66 (40.5%) displayed a shift in heteroplasmy since they presented higher heteroplasmy levels in tumors than normal tissues in all positive patients, as exemplified by A1736G, T4248C, and C16290T (Figure 2 and Supplementary Figure 3). Variants such as A663G, A10398G, C10400T, C12705T, and T16189C showed a wide range of heteroplasmy levels among tumors (Figure 2).

### Distribution of Somatic Mutations

We detected 384 variants displaying MAF < 0.01 in blood samples, but >0.01 in tumor samples. The 211 variants showing MAFs between 0.001 and 0.01 in normal tissues were named as probable mutations, and the remaining 173 were identified as somatic mutations (<0.001 MAF in normal tissue). Although 14 (6.6%) of the probable mutations presenting very low MAF in normal tissue (<0.008) were homoplasmic in tumors and 47 (22.3%) of all probable mutations were detected in more than two breast tumors (being T8430A: 9.8% and A8439C: 6.5% the most frequent), toward reducing biases derived from the technology and bioinformatic tools used, our further analysis was focused only on those variants classified as somatic mutations.

All somatic mutations were found in 68 (73.9%) tumors, ranging from 1 to 17 mutations per sample (X̄ = 2 ± 3). Single-nucleotide variants comprised the major type of somatic mutations (98%), followed by deletions (1%), and insertions (1%). Transversions were less common than transitions (16 and 84%, respectively) (Figure 1B). Of the 173 somatic mutations, 102 (59%) were distributed along protein-coding regions, whereas 71 (41%) were found in non-coding regions (D-Loop, rRNA, tRNA, intergenic regions). The D-Loop region (12.7%) and *CO1* (10.9%), *16S* (9.2%), *12S* (8.1%), and *ND5* (8.1%) genes harbored more mutations than the other genes. Our data showed that 147 (85%) somatic mutations were singletons while the remaining 26 (15%) were in more than two cases, with G3219T (*16S*, 3.3%), T3631A (*ND1*, 3.3%), and A7124G (*CO1*, 3.3%) being the most frequent mutations.

**FIGURE 3:**
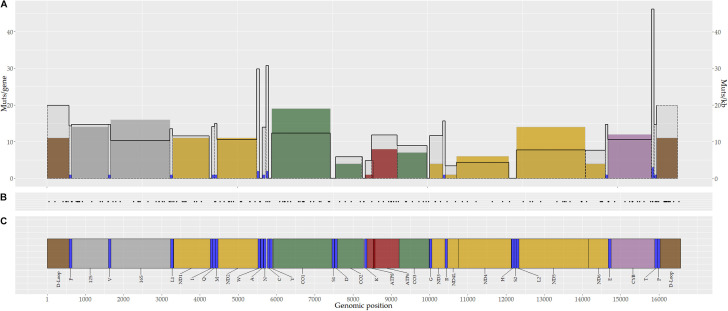
Genomic distribution of mitochondrial somatic mutations in breast tumors. A total of 173 somatic mutations were identified in breast tumors. **(A)** Number of somatic mutations by gene (color bars) and the mutation rate by gene (black continuous line). **(B)** Genomic position of mitochondrial somatic mutations. Each point corresponds to one mutation. **(C)** Mitochondrial genome map. Colors represent coding genes for protein complexes or non-coding regions (yellow, complex I; green, complex III; purple, complex VI; red, complex V; blue, tRNA; gray, rRNA; brown, D-Loop region).

Based on the mutation rate per kilobase (mut/kb), the *tRNA-T* (46.2 mut/kb), *tRNA*-C (30.8 mut/kb), and *tRNA*-W (29.8 mut/kb) genes, as well as D-Loop region (19.8 mut/kb), showed the highest mutation rates (Figure 3). Notably, no somatic mutations were identified in 10 tRNAs genes (I,A,Y,S1,D,K,G,H,S2, and L2) (Figure 3).

Regarding the heteroplasmy status of the somatic mutations, 37 (21.4%) were homoplasmic, whereas the remaining 136 (78.6%) showed a high range of heteroplasmy levels. The T15115C, C16270A, and C16527T mutations, which were detected in two patients, exhibited a homoplasmic status in one patient but heteroplasmic in the other one.

From all somatic mutations, 56 (32.4%) are not recorded in the MITOMAP database (41), including two of the most frequent mutations detected in our cohort (G3219T and T3631A).

### Functional Effect Prediction Analysis of Somatic Mutation

To determine whether the somatic mutations are either driver or passenger mutations, we annotated them using VEP software (42). Of 102 coding mutations, 34 (33.3%) were synonymous and 68 (66.7%) were non-synonymous (missense: 91.2%; non-sense: 5.9%; frameshift: 2.9%). Regarding non-coding mutations, 8 (8.4%) were located along regulatory regions, 46 (64.8%) within the rRNA and tRNA genes, and 19 (26.8%) fell into D-Loop and intergenic regions (Table 1). Given the nature of the mtDNA molecules, the T5726C and G5777A mutations (located in *tRNA*-N and *tRNA*-C genes, respectively) overlap in L-strand replication origin 1 (MT-OLR1) sequences and have a double function (Supplementary Table 3). Functional prediction analysis suggested that non-coding mutations (regulatory regions) might modify sequences associated with mtDNA replication and transcription processes (Table 2).

**TABLE 1:** Functional classification of the somatic mutations identified in breast cancer.

Effect type	Number of mutations, *n* (%)
Non-synonym	68 (66.7)
Missense	62 (91.2)
Non-sense	4 (5.9)
Frameshift	2 (2.9)
Synonym	34 (33.3)
Regulatory^*a*^	8 (8.4)
tRNA, rRNA	46 (64.8)
Intergenic	19 (26.8)

*^*a*^Two mutations in tRNA genes were overlapped with L-strand replication origin 1 (OLR1) and had a double function.*

**TABLE 2:** Somatic mutation previously associated with cancer.

Position	Gene	Consequence	Function	AA change	Frequency (%)*	Tumor type
G207A	D-Loop	Regulatory	LPS	–	1.1	Prostate, thyroid
T3394C	*ND1*	Coding	Missense	Y/H	1.1	Leukemia, colorectal, lung
T4823C	*ND2*	Coding	Synonym	V	1.1	Bladder, lung, head, and neck
T5567C	*TW*	Transcript change	tRNA	–	1.1	Endometrial, ovary
A5581G	Intergenic	Non-coding	Non-coding	–	2.2	Thyroid
C6371T	*CO1*	Coding	Synonym	S	2.2	Colorectal
T7389C	*CO1*	Coding	Missense	Y/H	1.1	Thyroid
G10290A	*ND3*	Coding	Missense	A/T	1.1	Endometrial
T10463C	*TR*	Transcript change	tRNA	–	2.2	Endometrial, thyroid, colorectal
G12630A	*ND5*	Coding	Synonym	W	1.1	Colorectal
G13333A	*ND5*	Coding	Missense	A/T	1.1	Breast
A13966G	*ND5*	Coding	Missense	T/A	1.1	Prostate
T14470C	*ND6*	Coding	Synonym	G	1.1	Colorectal
T15831C	*CYB*	Coding	Missense	I/T	1.1	Endometrial
A15836C	*CYB*	Coding	Missense	I/L	1.1	Breast
G15928A	*TT*	Transcript change	tRNA	–	2.2	Colorectal
G15995A	*TP*	Transcript change	tRNA	–	1.1	Endometrial
C16134T	D-Loop	Non-coding	Non-coding	–	1.1	Glioblastoma
A16162G	D-Loop	Regulatory	TAS	–	1.1	Ovary
A16163G	D-Loop	Regulatory	TAS	–	1.1	Ovary
C16248T	D-Loop	Non-coding	Non-coding	–	1.1	Ovary
C16261T	D-Loop	Non-coding	Non-coding	–	1.1	Nasopharyngeal, ovary
C16294T	D-Loop	Non-coding	Non-coding	–	2.2	Ovary
C16296T	D-Loop	Non-coding	Non-coding	–	1.1	Ovary
C16527T	D-Loop	Non-coding	Non-coding	–	1.1	Ovary, pancreatic

AA, amino acid; LPS, L-strand promoter sequence; TAS, termination associated sequence. *Frequency corresponding to population analyzed in this study.

### Somatic Mutations Associated With Breast Cancer and Other Cancer Types

To determine if the mtDNA somatic mutations identified in this study have previously been associated with cancer, all mutations were examined in the MITOMAP database. We found that 25 (14.6%) of these mutations have been identified in several types of human cancer. Only G13333A and A15836C mutations, which were detected in one patient (1.1%), have been recorded in breast tumors (Table 2).

### Distribution of Somatic Mutations by Breast Tumor Subtype

We assessed the association between mitochondrial mutations and clinical characteristics of breast cancer. No significant differences were found in terms of age, status, or tumor subtype among patients carrying any number of mutations and with no mutations (*P* > 0.05).

Unsupervised hierarchical clustering analysis with all 173 somatic mutations did not show any association between the mtDNA mutational profile and breast tumor subtypes. The distribution of all mutations and their heteroplasmy levels differed between patients (Supplementary Figure 4). The LA subtype displayed the highest mutation rate (5.97 mut/kb), followed by LB, TN, and H2 subtypes (1.32 mut/kb, 1.08 mut/kb, and 0.72 mut/kb, respectively). The LA subtype also showed the highest number of mutations (99/173), followed by LB (53/173), TN (18/173), and H2 (12/173) tumor subtypes. Most mutations were exclusively found in one tumor subtype (LA subtype: 88, LB: 44, TN: 17, and H2:7) (Supplementary Table 4). Eleven (A2641C, T3535A, G5777A, C6371T, G8292A, T10463C, A11221G, G15928A, C16150T, C16294T, and C16527T), and 2 (A15496C and A644C) mutations were detected in 2% of the LA and LB tumors subtype, respectively. Seven mutations were shared by LA and LB tumors subtypes, but no one mutation was present in all tumor subtypes (Supplementary Figure 5). No statistical significance was found in terms of the distribution of somatic mutations and breast cancer subtypes (*P* = 0.86; Supplementary Table 4).

### Tumor Mutational Burden Association and Overall Survival

To determine the use of mtDNA tumor mutational burden as a potential predictor of survival in breast cancer, a Kaplan–Meier analysis was performed (Supplementary Figure 6). Regarding OS, our analysis revealed no statistically significant differences among positive *versus* negative mtDNA somatic mutation groups (HR = 1.637; 95% CI = 0.401–6.683), or when comparing a low (≤2 mutations) or high (>2 mutations) mutational burden (HR = 1.385; 95% CI = 0.329–5.833). The threshold was established according to the mutational burden mean (*n* = 2) of somatic mutations.

## Discussion

Breast cancer, the leading cancer type in women around the world, is characterized by its complexity and its highly heterogeneous genetic background, resulting from the interaction between known environmental factors and mutations in the nuclear genome and mtDNA. The mitochondrial genome function is relevant for the transformation processes; tumor cells are able to escape from different regulatory mechanisms, but they cannot evade energy flow mechanisms. Thus, cancer cells could gain a functional mtDNA mutation as a strategy to adjust energy metabolism during adaptation to oncogenic conditions (44–46). To evaluate the landscape of somatic mutation in breast tumors and to determine whether the mtDNA mutational burden is correlated with the OS of breast cancer, we sequenced 92 whole mtDNA paired peripheral blood–tumor samples from Mexican women.

### Native American Haplogroups in Mexican Women With Breast Tumors

Seven different haplogroups were identified in our samples. The Native American haplogroups (A,B,C, and D) were more common than the major European (H and J) and African (L) haplogroups. Although the present study is limited by the selected population (women with breast cancer), we found similar frequencies of the A, B, and C haplogroups with those reported in the Mexican-Mestizo (mixed) populations and Mexican-Americans living in the United States (47, 48). Considering our findings and those previously published, the Native American haplogroups’ frequencies agree with the demographic history of the Mexican-Mestizo population that is composed of Native-American (53%), European (42%), and African (5%) ancestry (48, 49).

We found a higher frequency of the D haplogroup (12%) than in previously published works about the Mexican-Mestizo population (6.2%) (50, 51). The D haplogroup has been associated with predisposition to breast cancer in the Chinese population and experimental data revealed that the D5 branch haplogroup promotes tumorigenesis through AKT activation, mediated by a high concentration of reactive oxygen species (ROS) (52, 53). However, we need to include more patients and to perform a case–control analysis to determine whether the haplogroup D has clinical significance in Mexican women with breast cancer.

### High Heteroplasmy Levels of Germline Variants in Tumors and Heteroplasmy Shifting

In this study, we were able to distinguish acquired mutations from germline variants and to detect heteroplasmy shifting by analyzing normal-tumor matched tissues. On the one hand, <33% of the germline variants exhibiting low heteroplasmy levels (1–2%) were detected in more than two subjects, and 29.4% of singletons germline variants have been annotated in the public databases, suggesting that these could be rare variants. On the other hand, for several germline variants (as A1736C, T4248C, and C16290T), the mutant allele was enriched in tumors in comparison with normal tissues (Figure 2). A663G, a marker of haplogroup A, was the most common germline variant. No evidence of an association among haplogroup A, or this variant with breast cancer risk, has been documented. However, the 663G allele has been reported as associated with various mitochondrial diseases (54–57). Additionally, A663G has been found as a rare variant in papillary thyroid carcinoma and nasopharyngeal carcinoma (frequency <1%) (58, 59). *In silico* predictions suggest that the change of A > G could destabilize the structure of the rRNA by interrupting the hydrogen bond between U and A, then altering its function (54, 60). Nevertheless, its role in cancer development needs to be addressed.

The remaining common variants, T146C, A153G, A235G, A663G, A1736G, T4248C, A4824G, G8027A, C8794T, C16111T, T16189C, C16290T, G16319A, T16362C, and T16519C, are part of well-known mtDNA haplogroups and have been detected in some types of cancer such as prostate, ovarian, nasopharyngeal, thyroid, colorectal, gastric, and glioblastoma (58, 59, 61–67). Notwithstanding, to the best of our knowledge, only C16290T, which was identified in 14.1% of our cases, has been reported in breast tumors with a frequency of <1% (68).

Differences of heteroplasmy levels among tissues (blood <20%, tumors 1–95%) observed in this study could be biased by contamination by a different proportion of germline mtDNA during sequencing (69) or that the unregistered singletons germline variants showing <2% of heteroplasmy load arose during the genomic sequencing process (70). Our current strategy does not allow us to identify whether the heteroplasmy comes from the enrichment of mutated mitochondria or from mtDNA copy numbers (mtDNA-CN) variations. Diverse analyses have highlighted the great heterogeneity of the mtDNA-CN in different tissues and cancer types (71–74), but studies comparing mtDNA-CN among blood and normal tissue adjacent to the tumor are scarce and have found inconclusive results (28, 74–77).

mtDNA heteroplasmy is a normal condition of randomized processes and regulated homeostatic mechanisms, such as the mitochondrial segregation during the cell division, and mitochondrial fusion and fission process (78, 79). The changes in allelic frequencies have been suggested to be the response to selective pressures generated under physiological and pathological environmental conditions (17). However, it could be also as a result of changes in mtDNA-CN that occurred during the mtDNA replication (which is cell cycle independent) (80–82), or under oxidative stress induced by endogenous and exogenous factors (hormones, age, dietary, etc.) (83). The correlation among heteroplasmy and mtDNA-CN has scarcely been explored, and higher levels of heteroplasmy were correlated with lower mtDNA-CN in the central nervous system, but not in other tissues (73). In breast cancer, controversial results are found (83, 84), and it has been suggested that both processes are not interacting with each other and play different roles in the development of breast tumors (83). Although we have to consider that the shift toward homoplasmy observed in the present work could be influenced by mtDNA-CN rather than an enrichment of mutated mitochondria, heteroplasmy shifting has been reported in kidney and thyroid carcinomas (85), as well as breast cancer (28). Studies indicate that tumor cells show a neutral evolution of their mtDNA and even have the ability to tolerate pathogenic mutations; however, some mutant alleles reaching a critical threshold can contribute to cancer development and progression (85). The molecular mechanisms by which these alleles (whose heteroplasmy levels shift in the tumor) contribute to carcinogenesis is still unclear.

### High Frequency of Heteroplasmic mtDNA Mutations in Breast Tumors

In our study, 24 (26.1%) breast tumors were negative for somatic mutations and 68 (73.9%) were positive; over 78% of these mutations were heteroplasmic. Studies of breast cancer and diverse types of tumors have reported different proportions of tumors carrying mtDNA mutations (73.7 and 45.7%, respectively) (26, 28, 69, 86). The discrepancies among these findings could be partially explained by limitations derived from the sequencing methods, or due to the different thresholds used to define a somatic mtDNA mutation (28, 86). We found a high frequency of heteroplasmic mtDNA mutations and coding mutations (mainly non-synonym mutations), as well as a high mutation rate in the D-loop region and tRNA genes, as previously reported in several types of tumors (86, 87).

Coding mtDNA mutations could alter the protein function and OXPHOS system, and non-coding mutations might affect fundamental biological processes such as mtDNA replication, transcription, and structural mtDNA organization, thus conferring advantages for tumor cell proliferation (88, 89).

It has been suggested that deleterious pathogenic mutations, arising during the carcinogenic process are eliminated when protein function and viability cells are compromised, but selected if they enable cell proliferation (44, 90–93). In fact, it has been proposed that due to the *ND5* proton channel function, mutations in this gene might contribute to a deficient yield of the OXPHO system, leading to a high rate of oncogenic mutations in nuclear DNA and mtDNA (94, 95). Studies describing mutations in metastatic lesions, which were not found in primary tumors, support the hypothesis of the relevance of mtDNA mutations in cancer progression (90, 96). Li et al., by analyzing 26 types of cancer, proposed that regions and functional units are under negative and positive selection processes, suggesting the important role of mtDNA mutations in tumors (86).

Various authors stated that missense mutations of mtDNA are non-tumorigenic since they do not determine the impairment of the respiratory chain (28, 97). Likewise, since tRNA mtDNA pathogenic mutations are rarely fixed, the deleterious effect of these mutations might be functionally compensated for by gaining new mutations or altering mtDNA-CN (98). Other authors have proposed random processes defining the landscape of mtDNA mutations in cancer, suggesting that the heteroplasmic status arises in tumor progenitor cells by chance, without conferring any physiological or tumorigenic advantages (87). Primary tumors may have multiple clonal subpopulations that emerged during the numerous selective events required to complete the tumorigenesis process (99); thus, we have to consider that in addition to mtDNA-CN changes, clonal selection occurring during breast cancer progression could influence the levels of heteroplasmy observed in the present study and in other types of tumor (80, 100). More experiments are needed to understand the mechanisms underlying the mtDNA germline variants selection toward a homoplasmic state in breast cancer development.

As several of the recurrent mutations identified in this analysis (carried by >3% of our patients) have been related to other human diseases (58, 62, 101, 102), these mutations could also play a role in breast tumor development. However, it is necessary to validate our findings in an external cohort, including a population with a different haplogroup background. As well, we have to take into consideration that driver and passenger mutations may acquire oncogenic potential when, next to them, mtDNA gains more variants. In animal models, it was observed that the mtDNA containing two mutations in the *ND6* gene (G13997A and 13588insC) enhances the metastatic potential of tumor cells (12). Functional experiments could improve our understanding of the oncogenic biological processes mediated by these mutations (103, 104). The use of transmitochondrial hybrids might be helpful to address these issues (12).

The differentiation between a somatic mutation and a germline variant is a very complex task during the study of the mitochondrial genome. Technical and bioinformatics limitations might derive in misclassification of the variants. An interesting observation was the detection of the two coding variants T8430A (L22H) and A8439C (Q25P) as part of the probable mutation group. 8430A allele has been proposed to be associated with elevated ROS concentrations and reduced ATP synthesis (67, 105, 106). The 8439C allele has been described as a mutation in breast cancer and *in silico* analysis suggested that this mutation is highly deleterious and affects the *ATP8* function (107). Greater depth coverage of the mtDNA analysis could precisely determine the nature of the probable mutations (germline variants or somatic mutation) found in this work. As well, additional studies on T8430A and A8439C are needed to decipher their role in breast cancer development and progression.

### mtDNA Mutations and Clinical Relevance

In addition to the description of the landscape of the mtDNA mutations in breast tumors, we also evaluated their clinical relevance in tumor classification and prognosis. Over 85% of the somatic mutations were found with low frequency (<1%), probably due to individual variations (28). The LA and LB tumor subtypes displayed the highest number of mutations, sharing 4.1% of the mutations. However, we found neither an mtDNA mutation profile nor haplogroups associated with tumor subtypes. Few studies have explored the association between mtDNA mutational burden and haplogroups with breast tumor molecular subtypes, and their results are still inconclusive (51). Based on nuclear DNA mutation data showing that hormone-receptor-negative tumors have a higher mutational burden than hormone-receptor-positive tumors (108), we expected to observe the same behavior regarding mtDNA mutations. We suggest that the elevated mutational burden of hormone-receptor-positive tumors (LA and LB) could be explained by superoxide radical formation during estrogen metabolism, which promotes mtDNA alterations (103, 109). A recent study revealed a differential expression profile of mitochondria-related genes among molecular subtypes of breast cancer, perhaps derived from the partial contribution of the mtDNA mutations in breast tumor biology (110). Increasing sample size and balancing tumor subtypes could support our observations.

Overall survival analysis showed no differences among patients carrying a high *versus* low mtDNA mutational burden in tumors, which is in contrast with the data reported in breast cancer (28). A high mtDNA mutational burden has been associated with a worse outcome in pulmonary adenocarcinoma, acute myeloid leukemia, and pancreatic ductal adenocarcinoma (18, 111, 112); the opposite was observed in oral squamous cell carcinoma, and even acute myeloid leukemia, and breast cancer (28, 113, 114). We cannot exclude potential bias due to the nature of the studied mutations, the clinical characteristics of the patients included, or the ethnic/genetic background of the populations studied. Our small sample size and potential population stratification, since all patients were recruited in Mexico City, might also bias our results. As well, we cannot ignore the fact that other alterations, such as the mtDNA-CN (suggested as a potential prognostic biomarker for breast cancer), mito epigenetic processes, and nuclear genes that are involved in the mitochondrial biogenesis, may be clinically relevant (18, 71, 115, 116). Thus, studies are necessary for larger cohorts of patients to determine the significance of the mtDNA mutational burden as a biomarker in breast cancer.

This study describes the distribution of mtDNA germline variants and mutations in breast tumors in a population of Mexican women. We found a high mutation rate in the D-loop region and tRNA genes. Heteroplasmy analysis suggested that negative and positive selection processes are shaping the landscape of mtDNA mutations in breast cancer, but functional experiments are needed to further understand the oncogenic biological processes.

## Data Availability Statement

The original contributions presented in the study are publicly available. This data can be found here: European Nucleotide Archive (https://www.ebi.ac.uk/ena) (Accession: PRJEB40354).

## Ethics Statement

The studies involving human participants were reviewed and approved by the Instituto Nacional de Medicina Genómica. The patients/participants provided their written informed consent to participate in this study.

## Author Contributions

SJ-M, CP-A, and AH-M: conceptualization and design. CP-A, VB-P, FB-A, LA-R, CD-R, FV-C, and AT-T: sample identification and clinical follow-up. CP-A, HT, LG-R, and FB-A: data analysis. SJ-M and AH-M: supervision. CP-A and SJ-M: writing of the original draft. All authors: manuscript revision and approval of the submitted version.

## Conflict of Interest

The authors declare that the research was conducted in the absence of any commercial or financial relationships that could be construed as a potential conflict of interest.

## Abbreviations

AKT: AKT serine/threonine kinase
ATP6: ATP synthase membrane subunit 6
ATP8: ATP synthase membrane subunit 8
BRCA: BRCA DNA repair associated
BWA: Burrows–Wheeler aligner
CN: copy number
CO1: cytochrome C oxidase subunit 1
CO2: cytochrome C oxidase subunit 2
CO3: cytochrome C oxidase subunit 3
CYB: cytochrome B
ER: estrogen receptor
GATK: genome analysis tool kit
H2: HER2 positive breast cancer subtype
HER2: human epidermal growth factor receptor 2
HR: hazzard ratio
IHC: immunohistochemistry
LA: luminal A breast cancer subtype
LB: luminal B breast cancer subtype
MAF: mutant allele fraction
mtDNA: mitochondrial DNA
mtDNA-CN: mitochondrial DNA copy number
ND (1–6): NADH dehydrogenase subunits (1–6)
ND4L: NADH dehydrogenase subunits 4L
OXPHOS: oxidative phosphorylation
PR: progesterone receptor
ROS: reactive oxygen species
rRNA: ribosomal RNA
SNV: single-nucleotide variant
TN: triple negative
tRNA: transfer RNA
VEP: variant effect predictor.
