# Supplementary material for: Mitochondrial DNA Mutation Analysis in Breast Cancer: Shifting From Germline Heteroplasmy Toward Homoplasmy in Tumors
Source: Front Oncol. 2020 Oct 27;10:572954. doi: 10.3389/fonc.2020.572954 (PMC7653098; doi:10.3389/fonc.2020.572954)
Supplement: Supplementary file 1 [file Data_Sheet_1.PDF]

Supplementary Material

Supplementary Figures

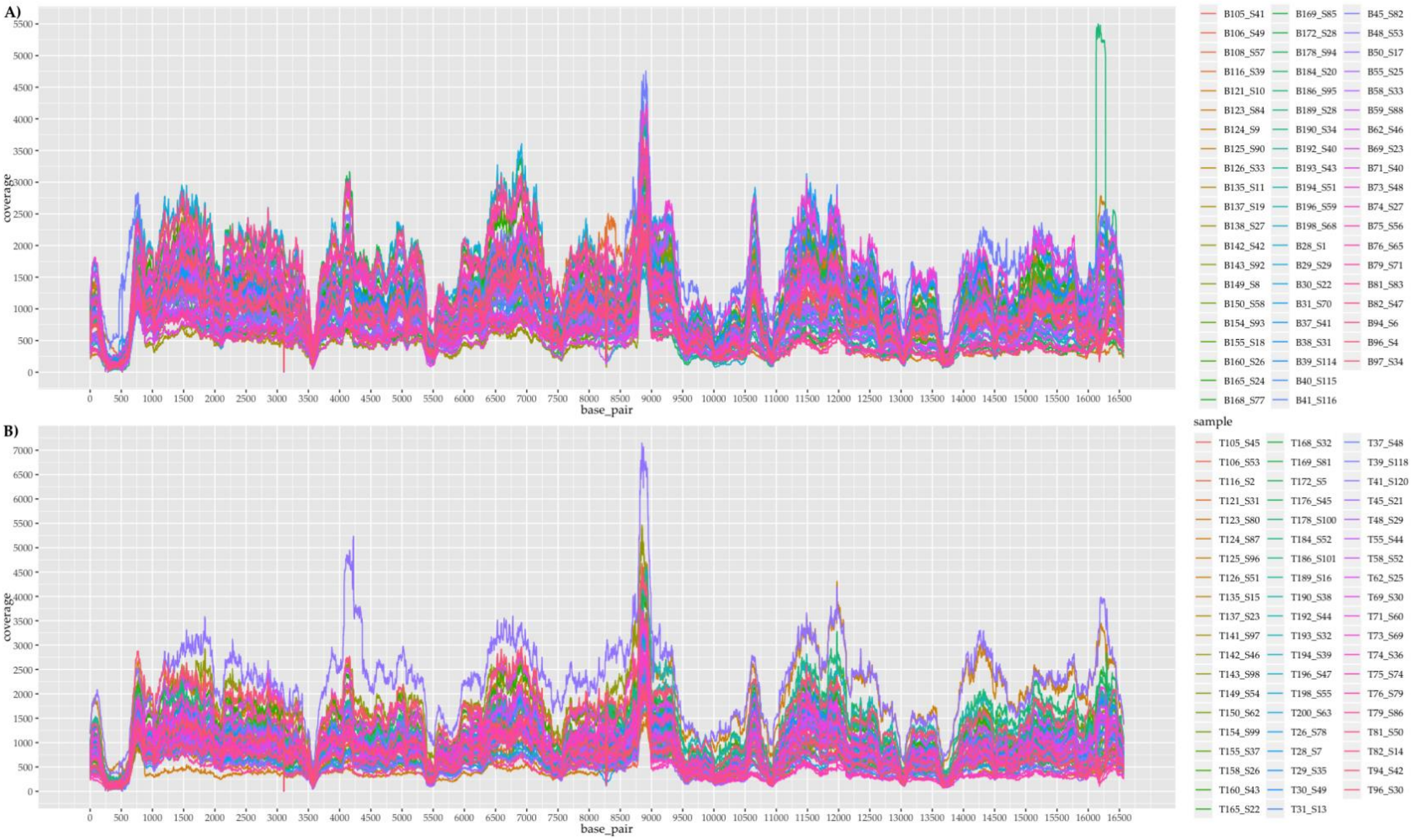

Supplementary Figure 1. Depth coverage of mitochondrial DNA sequencing. Total reads for each base position across all samples are showed. Each color line represents the depth coverage for one sample of blood (top graph) or tumor (bottom graph).

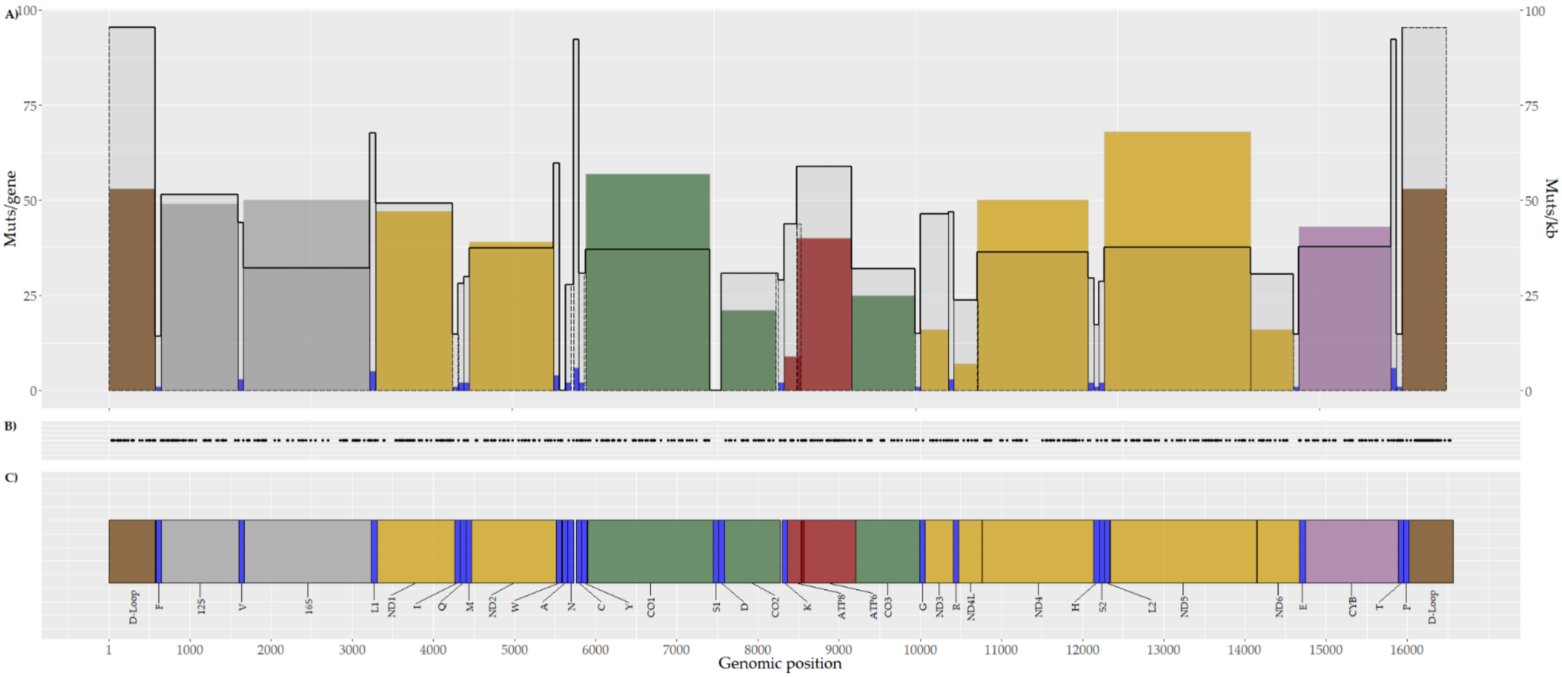

Supplementary Figure 2. **Genomic distribution of mitochondrial variants in breast tumors.** A total of 709 variants were identified in breast tumors. (**A**) Number of variants by gene (color bars) and the mutation rate by gene (black continuous line). (**B**) Genomic position of mitochondrial variants. Each point corresponds to one variant. (**C**) Mitochondrial genome map. Colors represent coding genes for protein complexes or noncoding regions (yellow, complex I; green, complex III; purple, complex VI; red, complex V; blue, tRNA; gray, rRNA; brown, D-Loop region).

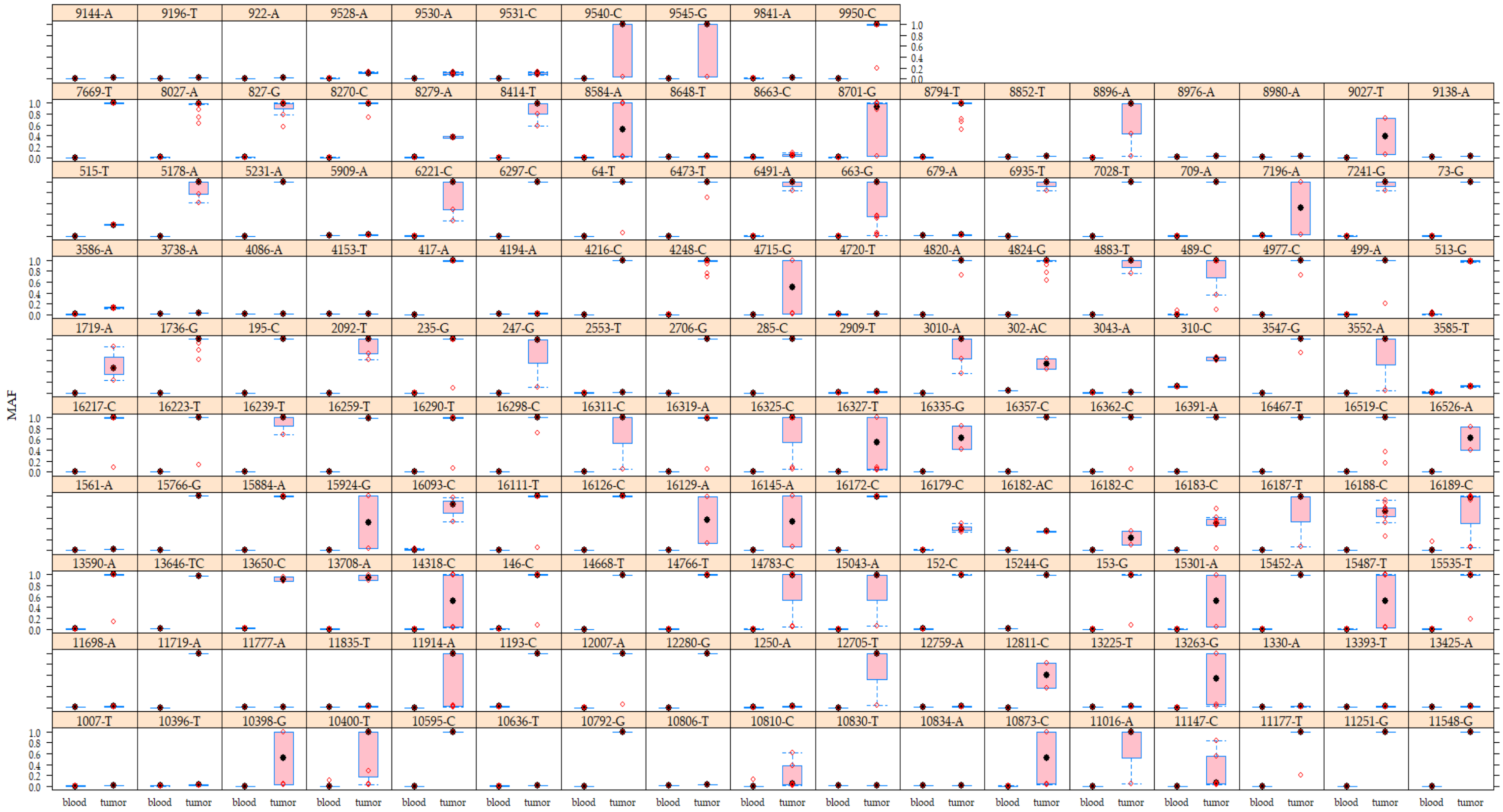

Supplementary Figure 3. Mutant allele fraction (MAF) of the 283 mitochondrial germline variants. MAF of germline variants in matched peripheral blood–tumor tissues. Higher MAF variability was observed in breast tumors than in blood samples**.** Each box displays the distribution of MAF values for each variant. Red circles represent patients.

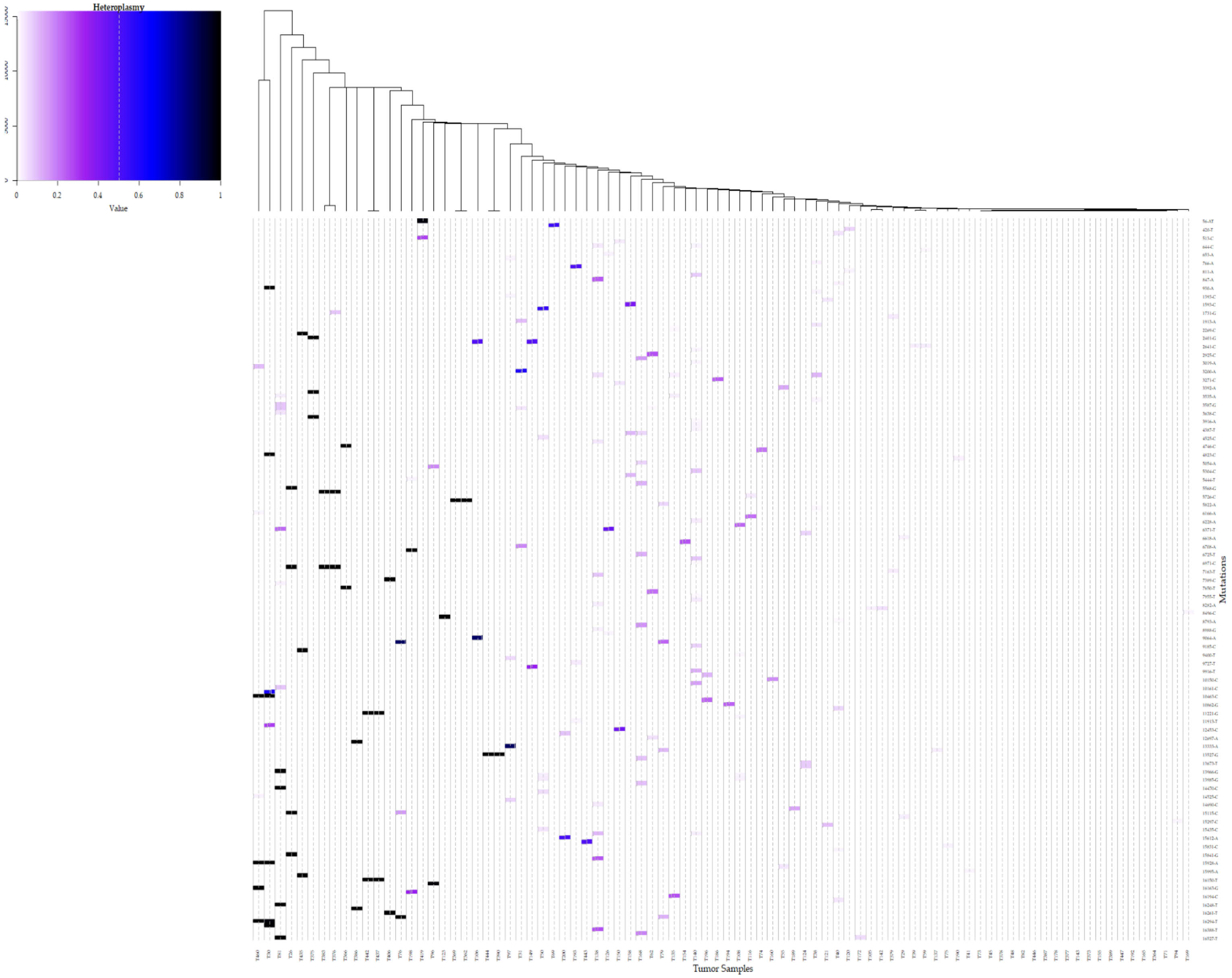

Supplementary Figure 4. Mitochondrial somatic mutations profiles in breast tumors. The heatmap represents unsupervised grouping of somatic mutations (rows) identified in each patient (columns). The color represents the proportion of the mutated allele fraction (MAF); white: ≥1.1%, black: 100%.

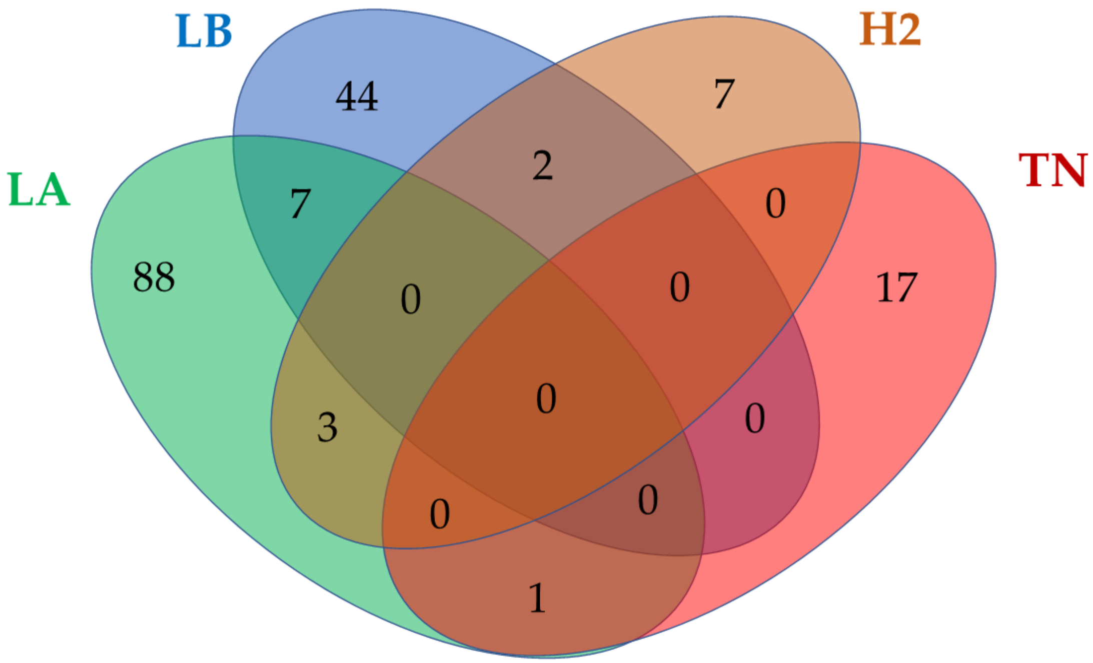

Supplementary Figure 5. Distribution of mutations in the molecular subtypes of breast cancer. The number of exclusive or shared mutations among the molecular subtypes is shown. Molecular classification was obtained using immunohistochemical markers of 90 breast tumors. LA: luminal A, LB: luminal B, H2: HER2, B: basal, TN: triple negative.

A)

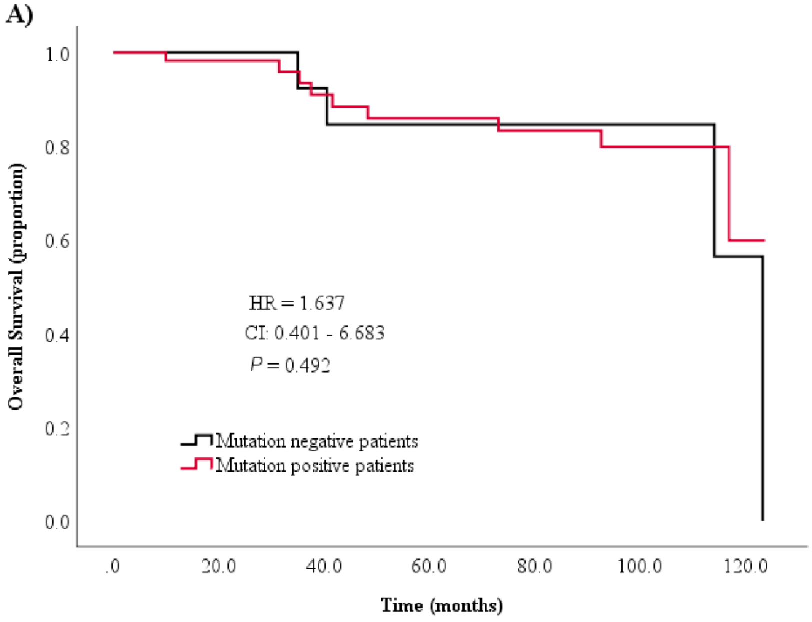

B)

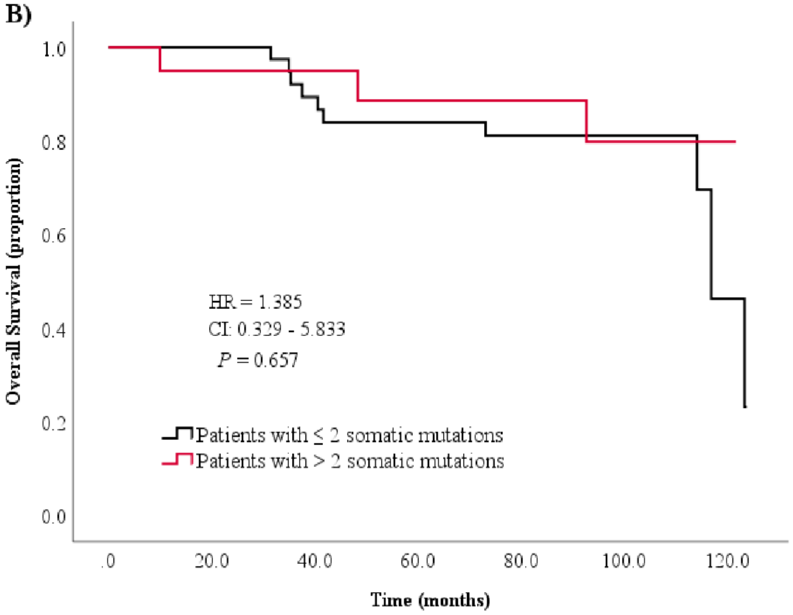

Supplementary Figure 6. Overall survival (OS) and mitochondrial DNA somatic mutation in breast cancer. Kaplan–Meier curves for OS were stratified by (**A**) presence/absence of somatic mutations and (**B**) low/high somatic mtDNA mutational burden. The threshold was established according to the mutational burden mean (n = 2) of somatic mutations. Log rank test P-values and Cox

proportional hazard model are provided, adjusted for age, age plus stage, and age plus stage and hormone receptor status.
